# Mycosporine-like amino acid and aromatic amino acid transcriptome response to UV and far-red light in the cyanobacterium *Chlorogloeopsis fritschii* PCC 6912

**DOI:** 10.1038/s41598-020-77402-6

**Published:** 2020-11-26

**Authors:** Carole A. Llewellyn, Carolyn Greig, Alla Silkina, Bethan Kultschar, Matthew D. Hitchings, Garry Farnham

**Affiliations:** 1 Faculty of Science and Engineering, Swansea University, Swansea, SA2 8PP UK; 2 Medical School, Swansea University, Swansea, SA2 8PP UK; 3 Faculty of Medicine and Dentistry, Plymouth University, Plymouth, PL4 8AA UK

**Keywords:** Biochemistry, Chemical biology, Plant sciences, Ecology, Environmental sciences

## Abstract

The “UV sunscreen” compounds, the mycosporine-like amino acids (MAAs) are widely reported in cyanobacteria and are known to be induced under ultra-violet (UV) light. However, the impact of far red (FR) light on MAA biosynthesis has not been studied. We report results from two experiments measuring transcriptional regulation of MAA and aromatic amino acid pathways in the filamentous cyanobacterium *Chlorogloeopsis fritschii* PCC 6912. The first experiment, comparing UV with white light, shows the expected upregulation of the characteristic MAA *my*s gene cluster. The second experiment, comparing FR with white light, shows that three genes of the four *mys* gene cluster encoding up to mycosporine-glycine are also upregulated under FR light. This is a new discovery. We observed corresponding increases in MAAs under FR light using HPLC analysis. The tryptophan pathway was upregulated under UV, with no change under FR. The tyrosine and phenylalanine pathways were unaltered under both conditions. However, nitrate ABC transporter genes were upregulated under UV and FR light indicating increased nitrogen requirement under both light conditions. The discovery that MAAs are upregulated under FR light supports MAAs playing a role in photon dissipation and thermoregulation with a possible role in contributing to Earth surface temperature regulation.

## Introduction

Cyanobacteria, the first evolutionary extant photosynthetic organisms, have evolved to adapt to a wide range of variable light environments and can survive exposure to both the ultraviolet (UV) and far-red (FR) ends of the visible light spectrum^1,2^. Exposure to natural levels of UV in cyanobacteria has been shown to have a wide range of physiological and biochemical effects, triggering changes in the photosystems, antioxidants and radical scavaging, and the induction of mycosporine amino acids (MAAs)^3,4^. Exposure to light at the FR end of the light spectrum has also been shown to have an extensive acclimatory affect on the genome of cyanobacteria^2^.

Commonly recognised for their UV sunscreening and photoprotection ability are a group of > 30 related low molecular weight aromatic amino acid derivatives called the MAAs. MAAs are a widely distributed in high-light marine, freshwater and terrestrial environments^5,6^. They are found in highest abundance within prokaryotic cyanobacteria and within the eukaryotic rhodophytes and dinoflagellates with highest concentrations often found in cells exposed to UV light^5,7,8^. Their absorption maxima range across the UV-A and UV-B regions from 310–360 nm with correspondingly high absorption coefficients (ɛ = 28,100–50,000 M^−1^ cm^−1^)^5,9^. Their strong UV absorption and photostability is not only important ecologically, but has led to an interest in MAAs as natural sunscreens to replace or supplement existing synthetic based sunscreens^10,11^. Other functions assigned to MAAs aside that of suncreening includes antioxidant activity and osmoregulation; these have been reported for the filamentous cyanobacteria species *Lyngbya* sp. CU2555^12^, *Nostoc* sp. R76DM^13^ and *Chlorogloeopsis fritschii* PCC 6912^6,14^.

The properties of mycosporine-glycine as an oxy-mycosporine amino acid are reported to be different to those of imino-mycosporines such as shinorine. In a study on *C. fritschii* PCC 6912 comparing UV and osmotic stress, UV-B was found to strongly enhance the accumulation of shinorine, whereas osmotic stress had a more pronounced effect on mycosporine-glycine^14^. In a study on the halotolerant cyanobacterium *Aphanothece halophytica*, mycosporine-2-glycine was found to be induced more under high salinity conditions than under UV-B^15^. In addition, mycosporine-glycine has been found to have strong antioxidant properties whereas MAAs substituted with two amino acids such as shinorine do not^5^. The ability of MAAs to absorb energy without producing reactive oxygen species also suggests that MAAs could have a role in photon-dissipation^16^.

As aromatic amino acid derivatives, MAAs have a core cyclohexenone or cyclohexenimine structure. These have a methoxy group at C-2 position and substitution at C-3 with an amino acid or an imino alcohol giving the oxy-mycosporines such as mycosporine-glycine; also a substition at C-1 with a second imino group leads to a wider group of imino-mycosporines including the commonly occuring shinorine. Understanding the biosynthetic pathways to the MAAs has been explored using the growing availability of genome sequences across many species of cyanobacteria, including for the more genetically complex filamentous species^8^. Comparative genome analysis on available sequenced genomes reveals that a four gene *mys* cluster (*mysA, mysB, mysC* and *mysD or E*), involving four steps leading to the production of MAAs is commonly present. The *mys* cluster has been found to occur commonly across filamentous cyanobacteria^17^.

The first biosynthetic step to MAAs is the production of desmethyl 4-deoxygadusol (DDG) and 4-deoxygadusol (4-DG). This can either occur via the shikimate pathway or more directly from sedoheptulose 7-phosphate^18,19^. In the shikimate pathway DHQ synthase (DHQS) catalyses conversion of DHQ to DDG, and in pentose phosphate pathway, desmethyl-4-deoxygadusol synthase (DDGS, a DHQS homolog) catalyses the production of DDG^17–19^. In the filamentous strain *Anabaena variabilis* ATCC 29413, both the pentose phosphate and shikimate pathways have been found to be linked to MAA biosynthesis^19^.

In the second step, the O-methyltransferase (MysB) converts DDG to 4-DG. In the third step, 4-DG incorporates the first nitrogen using an ATG grasp (MysC) to produce mycosporine-glycine, which is the branch point for all other MAAs. In the fourth step, mycosporine-glycine is converted to the imino-mycosporine, shinorine in *C.* *fritschii PCC 6912* and in *A. variabilis ATCC 29413*, with a non-ribosomal peptide synthetase (NRPS)-like enzyme (MysE)^18,20,21^, which contains adenylation, thiolation and thioesterase domains. The amino acid adenylation domain has the role of attaching the second amino group to the C1 position of mycosporine-glycine to produce the imino-mycosporine. In *Nostoc punctiforme* ATCC29133 and *Lyngbya* sp. PCC8106 a D-alanine D-alanine ligase homolog (MysD) replaces the NRPS-like MysE enzyme^22^.

The aromatic amino acids (AAAs) share biosynthetic pathways with the MAAs and are synthesised via the shikimate pathway with a branch point at dehydroquinate (3-DHQ). The shikimate pathway to production of AAAs is one of the most evolutionarily ancient metabolic pathways, and has been well studied in bacteria, algae and plants. It is estimated that > 30% of photosynthetically fixed carbon in vascular plants is directed from the shikimate pathway into tryptophan, phenylalanine and tyrosine^23^. Chorismate is the branch point for pathways to tryptophan, phenylalanine and tyrosine. AAAs are the building blocks for protein synthesis. They are also precursors to a wide range of essential end products such as pigments, hormones, vitamins and cell wall components, and serve as the precursors for the synthesis of many biologically and neurologically active compounds that are essential for maintaining normal biological functions^23^. For example, chorismate is required for the synthesis of folate (vitamin B9), tocopherols (vitamin E) and vitamin K. As a result AAAs are drug targets for human diseases including neurodegenerative diseases, schizophrenia, and cancers. Many of the downstream metabolites of AAAs are also important as natural products and in human medicine and nutrition^23^.

In particular, tryptophan is known to play a number of stress response and UV protective roles within plant and animal cells. Tryptophan is the amino acid precursor to serotonin and melanin. It has also been highlighted for its potential role in protection and cell signalling response regulator towards UV in plant cells. In *Arabidiposis*, tryptophan has been found to be part of a UV-B receptor known as UV-B resistence 8 (UVR8) serving as the UV-B chromophore which triggers a signalling pathway for UV protection^24,25^. Also involved in light and nutrient stress are the tryptophan-rich sensory proteins (TSPO); these are membrane proteins found across a range of organisms from bacteria to humans including filamentous cyanobacteria^26^. However, the regulation of tryptophan and other AAAs is still poorly understood.

The adaption of cyanobacteria to regions of the light spectrum aside from UV has also been studied. Red and green light adaption has been widely studied in terms of complimentary chromatic adapation (CCA). In CCA cells are able to rapidly adapt their photosystems (PSI, PSII) and phycobilisome (PBS) to red or green light by altering their complement of phycobiliproteins with green light absorbing phycoerthyrin or red light absorbing phycocyanin^2^. A major photo-acclimative response including the synthesis of FR absorbing pigments chlorophylls d and f have been observed in the filamentous cyanobacteria *Leptolyngbya* sp strain JSC-1 when transfered from white to red including FR light (> 700 nm)^27^. The FR absorbing chlorophylls *d* and *f* have also been observed in *C. fritschii*^28,29^. More widely, FR has been shown to induce non-photochemical quenching in the macroalgae *Ulva prolifera*^30^, and to protect against PSI photoinhibition in *Arabidopsis thaliana*^31^*.* However, we still know relatively little about how cyanobacteria regulate reponse to changing light conditions. More specifically there have been no studies to assess the response of MAA and AAA pathways under FR light in cyanobacteria.

Here we report two separate transcription profiling experiments focussing on the regulation of the MAA and AAA pathways in *C. fritschii* PCC 6912. *C. fritschii* PCC 6912 (Class Nostocaceae) is a Section-V filamentous strain originally isolated from soil in Allahabad, India^32^. *C. fritschii* is known to contain two MAAs, mycosporine-glycine and shinorine and these are inducible under UV^14^. In the first experiment, we explore the effects of supplementary low levels of UV-B (representing natural levels rather than higher and more damaging levels) on the MAA and AAA pathways. In the second experiment, we explore the effects of exposure to FR light on the same pathways. Both experiments use white light as the control. The experiments were carried out using different sequencing and bioinformatic techniques due to experiments being undertaken at different times and places. Whilst the results from the two experiments are not directly comparable they each provide unique insights into MAA and AAA pathways and the results from the FR light experiment reveals novel and unexpected findings.

## Results

**Figure 1:**
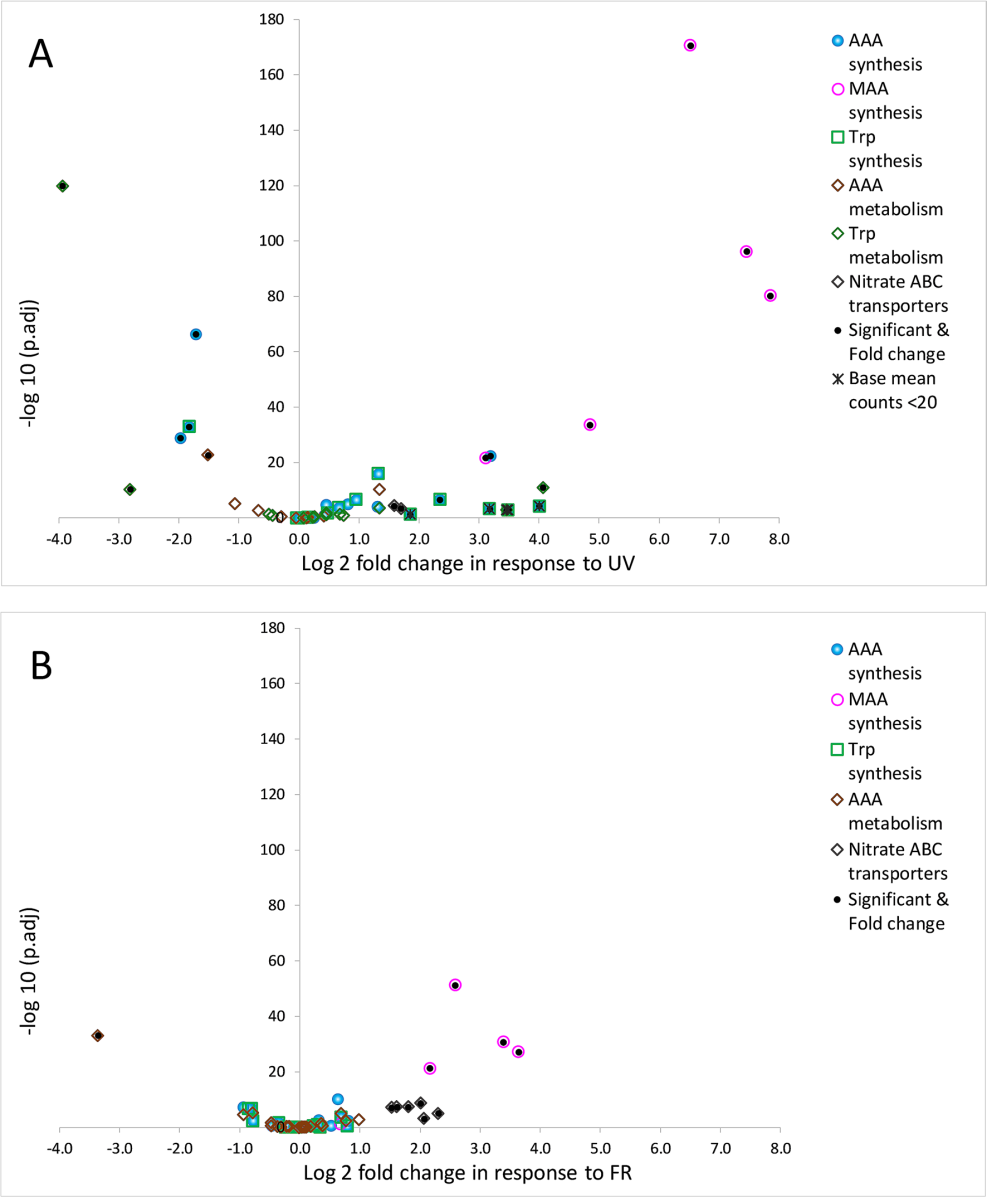
Volcano plots showing significance versus log2 fold change of expression in genes in *C. fritschii* PCC 6912 associated with exposure to (**A**) Low level UV-B and (**B**) FR Filled circles indicate significance with − 1.3 > log2 fold change > 1.3 *p *adj < 0.05.

The overall change in expression of genes in *C. fritschii* PCC 6912 exposed to UV-B and FR is shown in Fig. 1; this highlights the significant changes found associated with the MAA and AAA pathways. Associated details on the identified and significantly up and downregulated gene homologs associated with MAAs, AAAs and nitrogen transport are listed in Table 1.

**Table 1:**
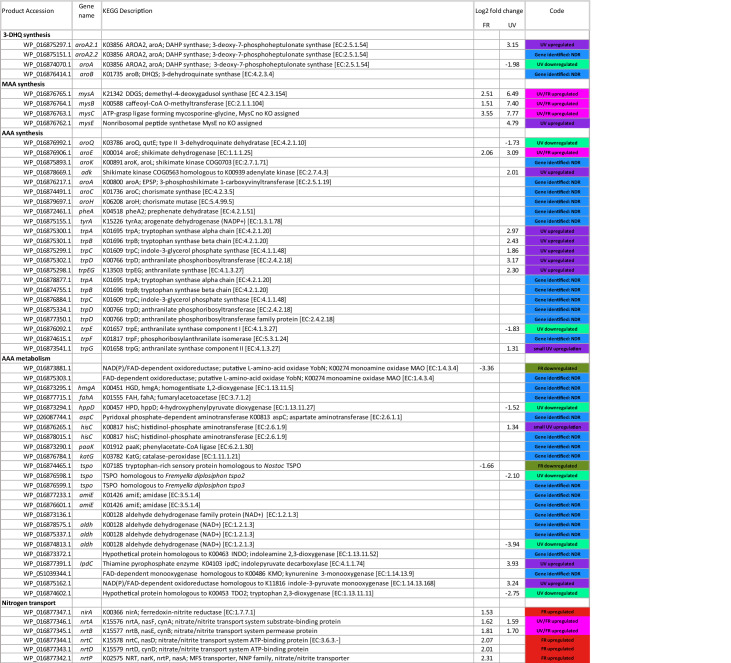
Transcriptional regulation of gene homologs associated with MAAs, AAAs and nitrogen transport in *C. fritschii* PCC 6912 exposed to UV and far-red light.

Log2 fold − 1.3 > log2 fold change > 1.3 and *p* adj < 0.05. Pink background UV and FR upregulated, purple UV-B upregulated, red FR upregulated, turquoise UV-B downregulated and green FR downregulated.

**Figure 2:**
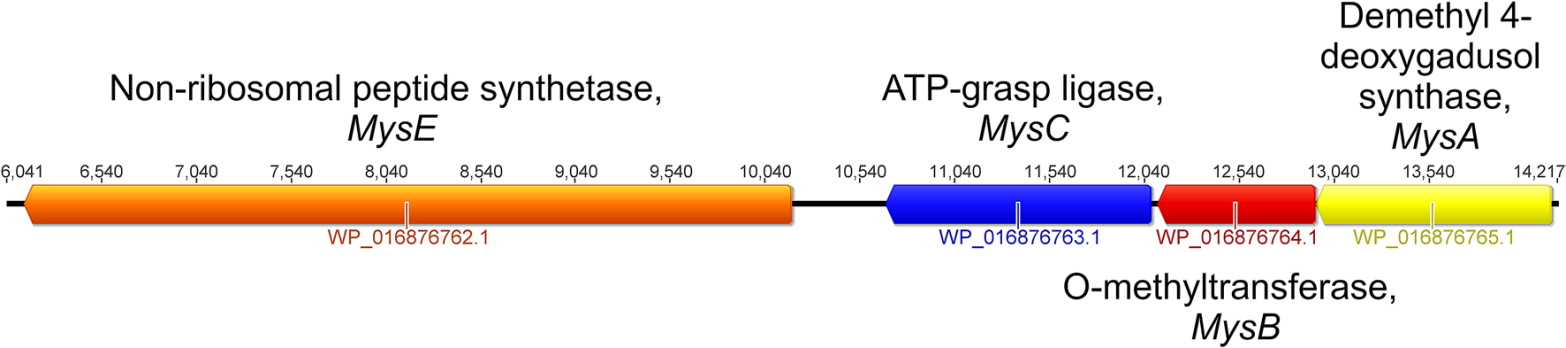
Gene cluster from *C. fritschii* PCC 6912. Cluster analogous to that found in *Anabaena variabilis* encoding the four-enzyme pathway for MAA biosynthesis. The position on NZ_AJLN01000152 is shown.

Most strikingly, from across the whole genone was that the MAA *mys* gene homolog cluster (Fig. 2) stood out as being significantly upregulated not only in *C. fritschii* PCC 6912 exposed to UV, but also when exposed to FR (Fig. 1). Other notable features included the gene homologs encoding nitrate ABC transporters, required to transport nitrogen into the cell, which were found to be significantly upregulated under both light conditions, and genes associated with the shikimate pathway to produce tryptophan which were predominately upregulated under UV-B but not affected by FR. Below we report these findings in more detail.

### Pathway to 3-dehydroquinate

**Figure 3:**
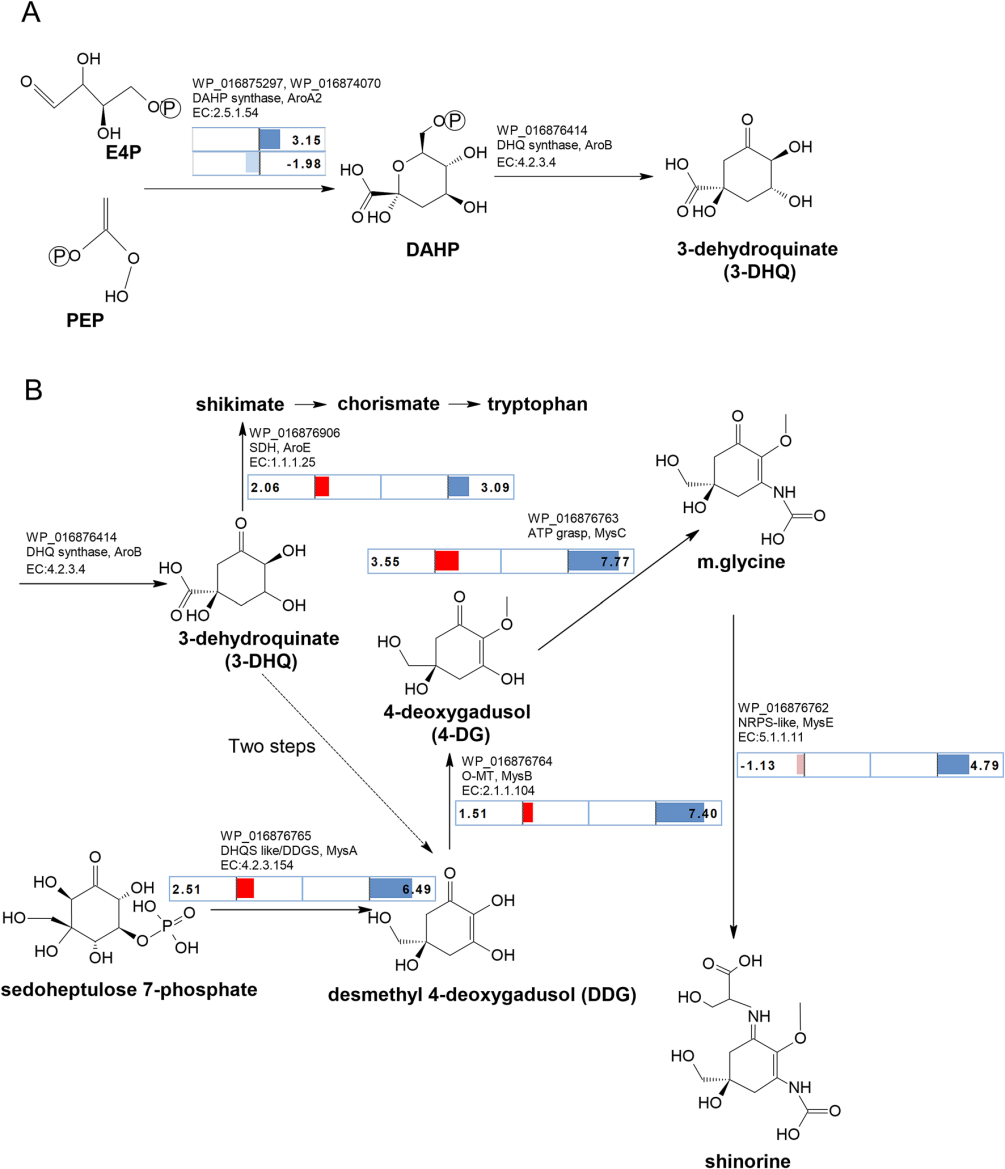
Regulation of genes on the MAA biosynthetic pathway in *C. fritschii* PCC 6912 under UV-B and FR light. Blue and red/pink represents significant log2 fold change for UV-B light exposure and FR light exposure respectively (− 1.3 > log2 fold > 1.3 and *p *adj < 0.05). Right and left of mid-line denotes upregulation and downregulation respectively. (**A**) Early common biosynthetic shikimate pathway to both MAAs and AAAs. Pathway from E4P and PEP to 3-dehydroquinate showing regulation under UV. E4P = D-erythrose 4-phosphate and PEP = phosphenolpyruvate; both intermediates in glycolysis and the pentose phosphate pathway. DAHP = 3-deoy-D-arabino-heptulosonate 7-phosphate. 3-DHQ = 3-dehydroquinate. (**B**) Pathway to MAAs via DDGS with change in regulation of the four *mys* gene cluster (WP_016876762—WP_016876765) under FR and UV. DHQS: 3-dehydroquinate synthase, DDGS: demethyl 4-deoxygadusol synthase, *mysA*; O-MT: caffeoyl-CoA O-methyltransferase, O-methyltransferase, *mysB*; ATP grasp, predicted ATP-dependent carboxylase. ATP-grasp ligase superfamily forming mycosporine-glycine, *mys*C (has no EC number); NRPS non-ribosomal peptide synthase—Amino acid adenylation domain, *mysE.* The branch point to AAAs from 3-DHQ is also shown.

3-DHQ is the branch point to producing both MAAs and AAAs. 3-DHQ is formed from phosphoenolpyruvate (PEP) and D-erythrose 4-phosphate (E4P), which are intermediates in glycolysis and the pentose phosphate pathway respectively (Fig. 3A). The first step is the production of 3-deoxy-D-arabino-heptulosonate 7-phosphate (DAHP) using DAHP synthase (AroA: E.C 2.5.1.54; Fig. 3A). In the second step, DAHP is converted to 3-DHQ using 3-dehydroquinate synthase (DHQS)^19,20,23^. We found three annotated DAHP synthase gene homologs in the *C. fritschii* PCC 6912 genome (Table 1). One DAHP synthase gene homolog was found within the chorismate-tryptophan cluster and was upregulated under UV light (WP_016875297: *aroA2.1*; UV log2 fold change of 3.15). Of the other two gene homologs, expression was downregulated under UV light for one (WP_016874070: UV log2 fold − 1.98), and unchanged for the other (Fig. 3A, Table 1). Expression of the single isolated DHQS gene was not affected by either lighting regimen (WP_016876414; EC 4.2.3.4; *aro*B).

### MAA synthesis

We found that the gene homologs representing the *mys* cluster (Fig. 2) encoding DDGS (MysA), O-methyltransferase (MysB) and the ATP grasp (MysC) and NRPS (MysE) were all upregulated in the UV-B experiment (WP_016876765 to WP_016876762; log2 fold change of between 4.79 and 7.7) (Fig. 3B; Table 1). However, most remarkably, we also found upregulation of *mys A*, *B* and *C* gene homolog cluster in the FR experiment. Here, however the upregulation was not as high as observed in our UV-B experiment (log2 fold change of between 1.51 and 3.55) (Fig. 3B). In contrast to the pattern seen under UV-B, under FR the fourth gene homolog in the MAA cluster, *mys*E, which catalyses the conversion of mycosporine-glycine to shinorine was slightly downregulated (WP_016876762; log2 fold change − 1.13) although this was not deemed as significant (Fig. 3B). The induction of MAAs under UV light has previously been established in *Gloeocapsa* sp and in *C. fritschii* PCC 6912^14,33^, however this is the first time that is has been observed in cells exposed to FR light.

**Figure 4:**
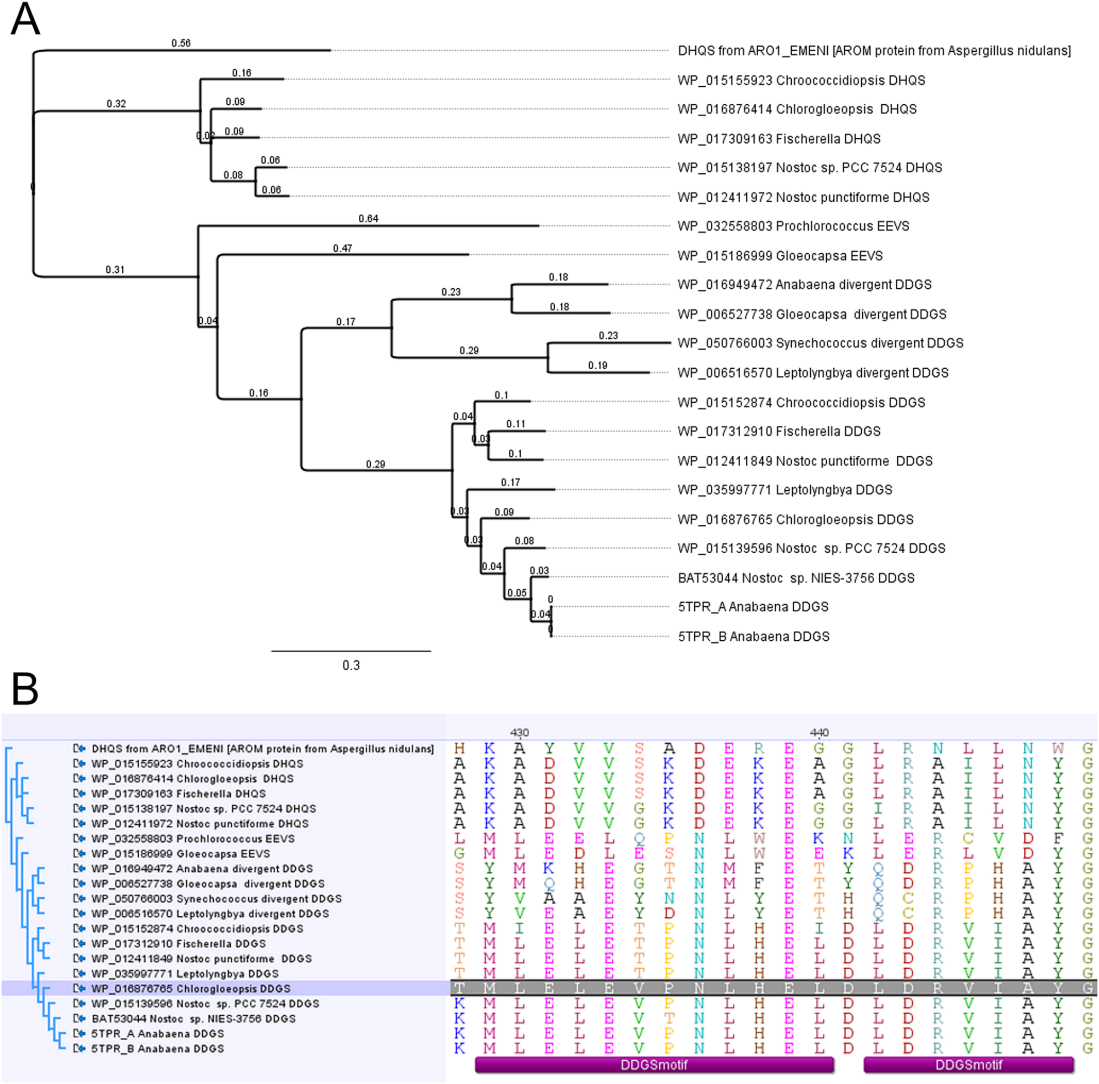
Analysis of sugar phosphate cyclase DHQS and DGGS sequences in *C. fritschii PCC 6912*. Comparative analysis with representative cyanobacterial proteins, including examination of the published discriminatory motifs^36^. (**A**) Neighbour Joining tree (using the Jukes Cantor a bootstrap of 1000) drawn in Geneious version 8.1.9 (https://www.geneious.com), from a MUSCLE alignment of cyanobacterial DDGS, EEVS and DHQS using the DHQS from the AROM protein of *Aspergillus nidulans* as an outgroup. (**B**) Alignment showing the DDGS discriminatory motif. Two proteins were identified: a DGGS [WP_016876765.1] (highlighted) and a DHQS [WP_016876414.1].

The DHQS-like gene homolog predicted as DDGS associated with the MAA cluster was upregulated under both FR and UV (WP_016876765; UV/FR log2 fold change 2.51 and 6.49 respectively; Fig. 3B). This DHQS-like (DDGS) gene homolog has been designated as *mysA*^17^. Two copies of DHQS have also been reported for *Anabaena variabilis* PCC 7937 and *Anabaena* sp. PCC 7120 where they were considered to be catalysing different reactions including one for MAA synthesis and the other for AAA synthesis^34^. We now know that one of these DHQS copies was most likely a DDGS^18^. Annotation of these sugar phosphate cyclase family proteins was confounded by DHQS sharing significant sequence similarity to 2-ep-5epi-valionone synthase (EEVS) and making current database annotation unreliable^35^*.* To confirm the identity of the sugar phosphate cyclase family proteins in *C. fritschii* PCC 6912, we used BLAST with representative cyanobacterial proteins, alignments and examination of the published discriminatory motifs^36^. From this we confirmed WP_016876765 as DGGS and WP_016876414a as DHQS (Fig. 4). There were no further sugar phosphate cyclase family proteins present.

High performance liquid chromatography (HPLC) analysis of the FR experimental biomass confirmed increases in MAAs under FR with a significant increase in mycosporine-glycine (Table 2; Supplementary Fig. S1). An increase was also observed for shinorine but because of variability across the three experimental replicate samples, this was not deemed to be significant. HPLC analysis of the UV-B samples revealed levels under the white light control to be below detection limit with low levels of shinorine and mycosporine-glycine induced in the samples corresponding to the transcriptome experiment (Table 2). We also observed that in additional samples collected at 4 h in the UV experiment, MAA levels were much higher at this time point, indicating the dynamic nature in acclimation of cells to the stress environment.

**Table 2:** Estimated average concentrations of MAAs (µg g^−1^dry wt): averages determined from three experimental replicates corresponding to replicate transcriptome experiment samples where *C. fritschii* PCC 6912 was exposed to either UV or Far Red (FR) light both with a white light control.

	Shinorine	Std.Dev	*p*	m-glycine	Std.Dev	*p*
**UV-B experiment**						
White light	0.0	0.0		0.0	0.0	
4 h	65.8	17.4	0.02	57.7	7.0	0.004
4h4d	1.4	1.7	0.3	0.6	1.0	0.42
**FR experiment**						
White light	21.0	0.3		286.0	29.0	
Far-red	42.0	19.0	0.2	423.0	56.0	0.03

In addition to the 4h4d transcriptome samples further HPLC samples were collected in the UV experiment at 4 h. HPLC method for UV experiment was according to Carreto et al.^37^and for FR experiment as described in the text. Concentrations estimated from HPLC peak areas and published extinction coefficients^38^. *p* value significance determined using a two tailed T test. For representative chromatograms for FR samples see Supplementary S2.

### AAA synthesis

**Figure 5:**
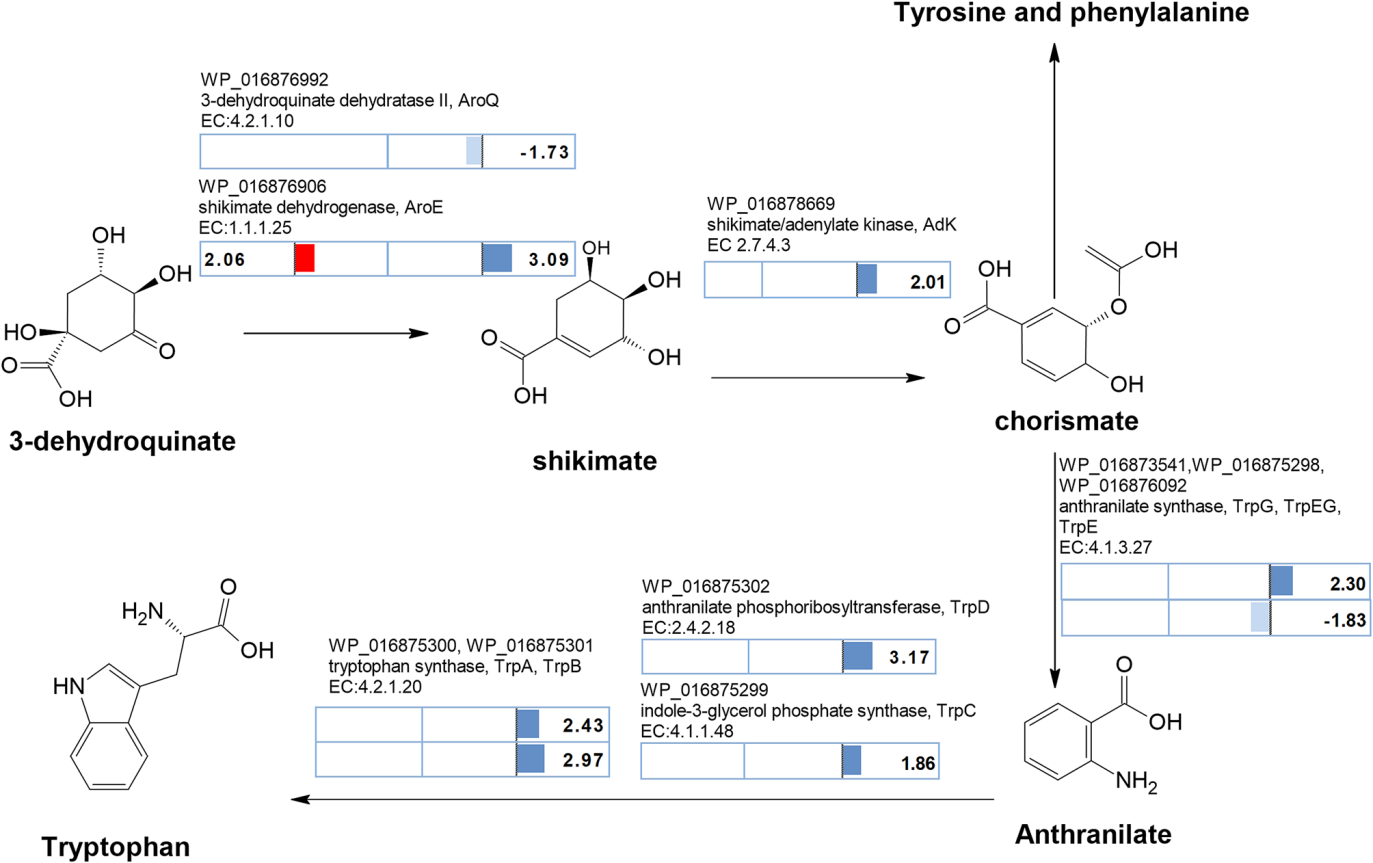
Shikimate pathway from 3-dehydroquinate to the aromatic amino acid tryptophan. Pathway adapted and simplified from Maeda and Dudareva^23^ with overlay of log2 fold change (− 1.3 > log2 fold > 1.3 and *p *adj < 0.05) in regulation in *C. fritschii* PCC 6912 under both FR (red denotes upregulation, no downregulation for this pathway) and UV (blue denotes upregulation, pale blue downregulation). No significant change in regulation for other key enzymes including 5-enolpyruvylshikimate 3-phosphate synthase and chorismate synthase involved in conversion of shikimate to chorismate. No change in regulation from chorismate to tyrosine and phenylalanine.

From 3-DHQ, 3-dehydroquinate dehydratase (DHD, EC:4.2.1.10) and then shikimate dehydrogenase (SDH, EC 1.1.1.25) catalyse the two steps to produce shikimate (Fig. 5). In *C. fritschii* a gene homolog (aroQ) encoding DHD was downregulated under UV and unaltered under FR (WP_016876992; UV log2 fold change of − 1.73). In contrast a gene homolog encoding shikimate dehydrogenase in *C. fritschii* to produce shikimate was significantly upregulated under both UV and FR (WP_016876906; EC.1.1.1.25; *aroE*; UV/FR log2 fold change of 3.09 and 2.06 respectively: Table 1).

Shikimate is converted to chorismate in a further 3 steps, via shikimate kinase, EPSP synthase and chorismate synthase. We found a bacterial shikimate kinase which was unaltered (WP_016875893.1: AroK). We also found an adenylate kinase family shikimate kinase, similar to those found in *Nostoc* and *Anabaena*, which was transcriptionally upregulated under UV (WP_016878669.1: AdK: UV log2 fold change 2.01 (Fig. 5, Table 1). There were no changes in transcriptional regulation under UV or FR for chorismate synthase or for 5-enolpyruvylshikimate-3-phosphate (EPSP) synthase. Chorismate is the branch point to the AAAs tryptophan, tyrosine and phenylalanine, as well as folate (B9) and to phylloquinone (vitamin K) involving multiple steps for each pathway (Supplementary Fig. S2). From chorismate, anthranilate synthase produces anthranilite on the pathway to produce tryptophan and indole or chorismate mutase produces prephenate as the branch point to tyrosine and phenylalanine. We found an upregulation of gene homologs under UV encoding enzymes for the conversion of chorismate to tryptophan; these were found in a gene cluster (WP_016875298 to WP_016875302; *tyrA*, *B*, *C* and *D*; UV log2 fold change 1.86–3.17) (Fig. 5). There was no change in expression in this gene cluster under FR (Fig. 5). It was within this cluster that we found DAHP which was, as indicated above, upregulated only under UV (WP_016875297; EC.2.5.1.54, aroA.2.1; UV log2 fold change 3.15) (Fig. 3).

### AAA metabolism

For the tyrosine and phenlyalanine metabolic pathways, there was little change in regulation. A putative monoamine oxidase potentially involved in tyrosine and tryptophan metabolism was downregulated in FR (WP_016873881.1; EC: 1.4.3.4; FR log2 fold change − 3.36). Additionally, 4-hydroxyphenylpyruvate dioxygenase in the tyrosine and phenylalanine metabolism pathways was slightly downregulated under UV (WP_016873294.1, EC:1.13.11.27; *hppD*, UV log2 fold change − 1.52) (Supplementary Figs. S2, S3). A reduction in tyrosine and phenylalanine in *C.* *fritschii* PCC 6912 exposed to UV-B has previously been observed^39^.

Next we investigated the tryptophan pathways. We first looked for a change in regulation associated with any tryptophan-rich sensory translocator protein (TSPOs). TSPOs are known to be associated with stress adaptation and have recently been found to be upregulated by green light and in response to nutrient defficiency and stress in the cyanobacteria *Fremyella diplosiphon*^26,40^. We found two similar adjacent TSPOs in *C. fritschii* with significant homology (> 75%) to TSPOs in the cyanobacteria *Fremyella displosiphon.* Expression was not changed for the gene similar to *Fremyella tspo*2, but the *tspo*3 homolog was moderately downregulated under UV-B and not affected by FR (WP_016876598; UV log2 fold change − 2.10). A third, less related TSPO homologous to proteins found in *Nostoc* sp. PCC7120 and *Anabaena variabilis* was downregulated under FR and unchanged under UV (WP_016874465; FR log2 fold change − 1.66). These TSPOs were thus differentially modulated under different light regimens.

Next we investigated if UVR8. Tryptophan has been associated with UVR8 protein serving as the UV-B chromophore that triggers a signalling pathway for UV protection^24^. We found no homolog to UVR8 in *C. fritschii.* Finally, we were interested to determine if there were changes in the tryptophan related indole acetic acid (IAA). There is some indication that the phytohormone related IAA is modulated by tryptophan and light^41^. One of the pathways to IAA is via indolepyruvate. Conversion of indolepyruvate to indoleacetaldehyde by indolepyruvate decarboxylase (IpdC) is the rate-limiting step in this pathway to IAA synthesis. We found indolepyruvate decarboxylase was upregulated under UV-B but not FR (WP_016877391: E.C 4.1.1.74; IpdC; UV log2 fold change 3.93) (Table 1; Supplementary Fig. S4). We also found upregulation under UV of a gene homolog involved in the production of indole-3-pyruvate monooxygenase which is also involved in IAA synthesis (WP_016875162: E.C.1.14.13.168; UV log2 fold change 3.24). In contrast, two gene homologs involved in tryptophan metabolism and formation of kynurenine and niacin using tryptophan 2,3 dioxygenase were downregulated under UV (WP_016874813; EC: 1.2.13; UV log2 fold change − 3.94 and WP_016874602; EC 1.13.11.11; UV log2 fold change − 2.75 respectively).

### The role of nitrogen

Nitrogen, with its major source being nitrate in cyanobacteria, is essential to the biosynthesis of MAAs and AAAs. We found that in addition to the *mys* cluster standing out for upregulation under FR, that the nitrate transport genes were also prominent for their upregulation under FR. Nitrate transporters activate transport of nitrate into the cell for glutamine production^42^. For AAAs, nitrogen is introduced with glutamine converting chorismate to anthranilate (Fig. 5). Glutamine, required for anthranilate production, utilises a source of nitrogen which is primarily sourced through nitrate in the growth medium. Glutamine is required to introduce nitrogen into the aromatic chorismate ring structure converting it using anthranilate synthase into anthranilate on the pathway to tryptophan (Fig. 5). For MAAs, nitrogen is added to 4-deoxygadusol using an ATP grasp (Fig. 2). Tyrosine and phenyalanine require additional conversion before nitrogen is added via aminotransferases^23^. Nitrate transporter genes, *nrtA* and *nrtB* were found within an nitrogen regulation cluster and were upregulated under both UV and FR (WP_016877346-WP_016877345; log2 fold change range 1.59–1.81). Associated *nirA*, *nrtC*,* nrtD* and *nrtP* were upregulated only under FR (WP_016877347-WP_016877342 FR log2 fold change 1.53–2.31) (Table 1). Our results showing upregulation of genes associated with nitrate assimilation indicates that increased nitrogen is required under both UV and FR light conditions.

## Conclusion

MAAs and AAAs are two groups of low molecular weight aromatic compounds containing nitrogen that are related in their biosynthetic pathways. MAAs are widely recognised as being important in photoprotection and AAAs are important in the synthesis of proteins and other essential end products. MAAs in addition to their important physiological role and evolutionary and ecological relevance are increasingly sought after for their use in sunscreen and cosmetic products. Likewise, in addition to their important physiological role, AAAs have a wide application in human health and more widely in human medicine and nutrition.

Our results provide new understanding on the regulation of MAA and AAA biosynthetic pathways under low level UV and FR light. Most importantly, we have shown for the first time that the *mys* gene homolog cluster in *C. fritschii* PCC 6912 is associated with MAA synthesis up to mycosporine-glycine is upregulated when exposed to UV and to FR light. Increases in MAAs measured using HPLC was found in extracts of the corresponding samples of *C. fritschii* PCC 6912 exposed to FR.

Our finding that there is upregulation of the MAA pathway under FR suggests that MAAs may have a role in the photon dissipation of light and thermodynamic optimisation. Recently scytonemin, a related UV-A sunscreen compound, was shown to increase soil temperature in cyanobacterial biocrust communities, decreasing soil albedo significantly with the potential for impacting biosphere feedback and affecting the climate^43^. The ability of FR to influence production of MAAs supports the role of these compounds as photon dissipators opening up new possibilites on the importance of these compounds in heat regulation on Earth.

We also found a cluster of genes associated with the pathway to tryptophan upregulated under UV but not under FR. Pathways to tyrosine and phenylalanine biosynthesis were unaltered under both UV and FR. Nitrate transporters were also found to be upregulated under both UV and FR light with some only being upregulated under FR indicating the requirement for additional nitrogen. Our results highlight the complex finely tuned interconnectivity between AAAs and MAAs and their response to a changing light environment under both UV and FR.

## Methods

### UV-B light exposure experiment

Experimental set up for the low level UV-B light exposure was as previously reported^44^. *Chlorogloeopsis fritschii* (Mitra) PCC 6912 was inoculated at 1:50 dilution from a master culture and cultivated in 5 L Erlenmeyer flasks containing 2 L BG11 media with 10 mM HEPES buffer at pH 7.5. The culture was perfused with 1% CO_2_ and was maintained at 38 °C using white light (410–750 nm) with an intensity of 60 µmol photons m^−2^ s^−1^ (Grolux fluorescent tubes). At exponential growth phase, cells were harvested and transferred to nine quartz Erlenmeyer 500 mL flasks containing 200 mL of fresh BG11, and 10 mM HEPES at pH 7.5 to give a concentration of 0.44 g L^−1^ wet weight (approximating to 0.04 g L^−1^ dry weight). All 9 flasks were exposed to the same white light as for the stock culture for 4 days and 4 h (100 h in total). Three flasks were exposed to white light with no UV-B light acting as the control (white) for 100 h, three flasks were exposed to white light supplemented with UV-B light for the final 4 h of the experiment (samples referred to as; 4 h) and, three flasks were exposed to white light supplemented with 4 h of UV-B light each day for 4 days (samples referred to as; 4h4d). Flasks exposed to UV-B light were placed 10 cm from UV-B tubes (Philips) supplying 3 μmol m^−2^ s^−1^ at wavelength range 300–310 nm (Supplementary S1).

At the end of the experiment, all 9 flasks were placed on ice and were centrifuged (3000 g) at 4 °C. The pelleted biomass was snap frozen in liquid nitrogen before storage at − 80 °C. Transcriptomics to determine differential gene regulation was undertaken on the white light (control) and 4h4d samples. HPLC analysis to determine MAA content was undertaken on the white, 4 h and 4h4d samples.

#### RNA preparation and sequencing

RNA was extracted with Trizol followed by terminator exonuclease digestion to enrich for mRNA and subsequently cleaned using a Qiagen RNeasy column. RNA sequencing was conducted at the Centre for Genomic Research, Institute of Integrative Biology at the University of Liverpool, UK, L69 7ZB, using the Life Technologies SOLiD sequencing platform. For each sample, at least 49,034,856 sequences were obtained (50 bp, min average quality 20 as per manufacturer specifications; per sample average sequence number: 57,516,996.44).

Alignment of reads was carried out using the *C. fritschii* PCC 6912 genome as reference. The sequences obtained for each sample were aligned on to the reference using Bowtie version 0.12.7, using the colour space option. Prior the alignment step, the sequences required the conversion to a pseudo-FASTQ file required as input for Bowtie. For each sequence, only the best alignment was reported by Bowtie, or one was randomly chosen if many were equally best. The average percentage of unambiguously aligned sequences was 47.67%, with a minimum of 35.9% and the maximum equal to 51.9. Considering the known 6968 genes, an average of 83.11% of these were identified as expressed across all the samples (ranging between 73.95% and 87.15%). All the obtained alignment files were processed using HTSeq-count^45^, and reads aligning to the reference genome sequences were counted according to the gene features that they mapped to, as defined in the GTF files.

The differential expression analyses between white and the 4h4d samples were performed using R (version 2.14) and edgeR package. The gene-counts were normalised using “loess smooth” method from the ‘limma’ package. The “GLM” model was applied to the normalised data (with EdgeR package), and the dispersion related to each gene (genewise dispersion) and the pairwise group comparisons were performed to identify differentially expressed genes for each of the three possible group comparisons. For each contrast, each gene with a *p* value below 0.05 (after adjusting for multiple testing effect using the False Discovery Rate approach^46^ were selected as differentially expressed for that contrast. Significant changes in regulation were defined as log2 fold change ≤  − 1.3 or ≥ 1.3 and *p *adj < 0.05.

### Far-red light exposure experiment

Three conical 1 L flasks containing 800 mL *C. fritschii* culture with an initial optical density (OD) at 750 nm of 0.1–0.2 Absorbance Units (AU) equating to 0.02–0.04 g L^−1^ dry weight were cultivated under white LED light with an intensity of 100 µmol photons m^−2^ s^−1^ for 6 days to an OD at 750 nm of 0.4 AU approximating to 0.08 g L^−1^ dry weight. 50 mL aliquots were taken from three flasks and transferred to 50 mL centrifuge tubes (Falcon) to provide the white light control samples. The flasks were then placed under FR LED light with emmision centred at 710 nm (Alibaba.com; Supplementary S1), providing ~ 18 µmol photons m^−2^ s^−1^, and grown for a further 24 h. After 24 h, 50 mL aliqouts were taken to provide the FR light samples (OD 750 nm of 0.5 approximating to 0.1 g L^−1^ dry weight).

#### RNA preparation and sequencing

Samples in centrifuge tubes were immediately cooled on ice then pellets for RNA extraction were prepared by centrifugation for 15 min at 4 °C at 3500 rpm, then further concentrated in a reweighed microfuge tubes at 4 °C at 5000 rpm. After supernatant removal, tubes were weighed on ice to calculate wet weights and the pellets were flash frozen in liquid nitrogen and stored at − 80 °C. Pellet wet weights were between 30 and 50 mg. For extraction, pellets were resuspended in 1 mL cold Trizol reagent (Thermo Fisher Scientific) and homogenised using 0.5 mm glass beads (VK05) in a Precellys 24 homogenizer (Bertin) at 6500 rpm for 2 × 20 s with a 10 s break. After 5 min incubation, the sample was extracted with 0.2 mL chloroform followed by centrifugation at 12,000×*g* for 15 min at 4 °C. The upper aqueous phase was mixed with an equal volume of 70% ethanol and applied to a PureLink RNA Mini Kit spin cartridge (Thermo Fisher Scientific). The sample was washed and treated on-column with PureLink DNAse (Thermo Fisher Scientific) according to the manufacturer’s instructions except for an extra wash step, before drying and elution in 100 µL RNase free H_2_0.

RNA concentrations were determined using a NanoDrop ND-1000 Spectrophotometer (Thermo Scientific). DNA and RNA concentrations were also measured separately using a Qubit 3.0 Fluorometer (Thermo Fisher Scientific). Residual DNA contamination was removed by two 30 min treatments at 37 °C using 1.5 µL TURBO DNase (Thermo Fisher Scientific) followed by enzyme removal using the inactivation reagent supplied in a TURBO DNA-free Kit (Thermo Fisher Scientific) according to the instructions. To monitor the presence of DNA, primers were designed to SecA, a protein translocase subunit suitable as a reference gene in the heterocystous cyanobacteria *Nostoc* sp. PCC 7120^47^ and realtime PCR was performed before and after treatment using the CFX96 Touch Real-Time PCR Detection System (Bio-Rad Laboratories) with PerfeCTa SYBR Green FastMix (Quantabio). This confirmed complete removal of amplifiable DNA from all samples. RNA purity and quality was confirmed by running on an Agilent 2100 Bioanalyzer using a RNA 6000 Nano Kit and by cDNA synthesis using qScript cDNA SuperMix (Quantabio) followed by realtime PCR amplification as above. Prior to analyses, adapter sequences were removed from sample reads using the cutadapt tool.

Total RNA was used as starting material for the generation of sequence ready libraries. Briefly, bacterial ribosomal RNA was removed from the samples with use of a RiboZero bacteria kit. To preserve strand specificity, rRNA free RNA was subjected to TruSeq stranded mRNA sample preparation. Final libraries were normalised to 4 nM prior to pooling. A final library concentration of 20 pM was used to sequence the libraries on a MiSeq platform at the Swansea University Sequencing Facility, generating a total of 99,448,410 high quality reads between all samples using multiple V3 2×75 bp PE sequencing runs^48^.

Due to an incomplete genome assembly and annotation of the *C. fritschii* genome, analysis of gene expression was carried out in two phases. Initially transcripts were de novo assembled using Rockhopper 2^49,50^ in order to identify novel transcripts absent from the current genome annotation due to the genome’s draft status (gene truncation due to contig boundary interruption of coding sequence causing absence of annotations). The de novo gene list was added to the current annotation. Reads for each sample were mapped to the draft genome using the Subread aligner and read summarization carried out by feature Counts using our improved annotation^51,52^.

Un-normalised Read count summarisation was fed into DeSeq2 R package^53^ for differential gene expression analysis measuring the effect of the two conditions, white light and red light, on gene expression levels. Log2 fold change along with Wald test *p* values and adjusted *p* values were generated from the DeSeq2 normalised dataset. Significant changes in regulation were defined as log2 fold change ≤ − 1.3 or ≥ 1.3 and *p *adj < 0.05.

### MAA extraction and analysis for UV_B and FR samples

Each pellet of a known weight was re-suspended in 100% HPLC grade MeOH (1 mL) and left in the dark at 4 °C overnight (24 h). After centrifugation (5 min at 12,000 rpm), the supernatant was removed and evaporated to dryness using a rotary vacuum concentrator. The dried extract was re-dissolved in 600 µL of deionised water and transferred to autosample vials for HPLC analysis^13^.

For the UV experiment, MAA HPLC analysis was according to Carreto et al.^37^ using a Thermo Accela Series HPLC system with a binary pump, chilled autosampler (4 °C) and photodiode array detector. Separation was with two columns connected series thermostated at 35 °C (1) a polymeric double-endcapped C18 column (5 μm, 4.6 mm i.d. × 150 mm length; Alltima, Alltech) and (2) a polymer-coated silica reversed-phase C18 column (5 μm, 4.6 mm i.d. × 250 mm length; CapCell Pak UG, Shiseido) protected with a guard column cartridge (4.6 mm i.d. × 20 mm length; Alltima, Alltech).

For the FR experiment, MAA HPLC analysis was performed using an Agilent 1100 system with a binary pump, an autosampler injector and diode array. The stationary phase was an Alltima Altech C18, 4.6 × 150 mm, 5 µm column thermostated at 35 °C. The re-suspended extracts were injected (100 µL) using an auto-sampler. The mobile phases consisted of; Eluent A: Water (0.01% TFA, v/v) and Eluent B: 70% methanol (0.054% TFA, v/v) with a gradient of; 99% A for 10 min, to 80% A over 5 min, to 1% A over 5 min, held for 3 min and increased to 99% A over 2 min. MAAs were monitored at 320 nm with spectral scanning of HPLC separated peaks from 250 to 400 nm. MAAs were identified based on spectral matching of the two main characterised MAAs (shinorine, λmax = 334 nm; m-gly, λmax = 310 nm) known to be present in *C. fritschii*^14^. Concentrations expressed as µg g^−1^dry weight were estimated from HPLC peak areas and published extinction coefficients using the Beer-lambert law using ε = extinction coefficient (shinorine, ε = 44,700 M^−1^ cm^−1^; m-gly, ε = 28,790 M^−1^ cm^−1^)^38^, *p* value significance was determined using a two-tailed t-test.

## Supplementary information

Supplementary Information.

## Data Availability

The datasets generated for this study can be found within an NCBI BioProject with Accession Number PRJNA545395.
