# Supplementary material for: Mycosporine-like amino acid and aromatic amino acid transcriptome response to UV and far-red light in the cyanobacterium Chlorogloeopsis fritschii PCC 6912
Source: Sci Rep. 2020 Nov 26;10:20638. doi: 10.1038/s41598-020-77402-6 (PMC7693272; doi:10.1038/s41598-020-77402-6)
Supplement: Supplementary file 1 — Supplementary Information. [file 41598_2020_77402_MOESM1_ESM.docx]

**Supplementary**

**Mycosporine-like amino acid and aromatic amino acid transcriptome response to UV and Far-Red light in the cyanobacterium *Chlorogloeopsis fritschii* PCC 6912**

Carole A Llewellyn^1^*, Carolyn Greig^1^, Alla Silkina^1^, Bethan Kultschar^1^, Matthew D Hitchings^2^, Garry Farnham^3^

1. Swansea University, Department of Biosciences, Swansea, SA2 8PP, UK

2. Swansea University, Medical School, Swansea, SA2 8PP, UK

3. Plymouth University, Faculty of Medicine and Dentistry, PL4 8AA, UK

* Corresponding author.

Email address: [c.a.llewellyn@swansea.ac.uk](mailto:c.a.llewellyn@swansea.ac.uk)

**Fig. S1**

Light spectra for the two light sources used in the two experiments. Blue - UV-B light source. Orange - FR LED light source.

**Fig. S2**

**
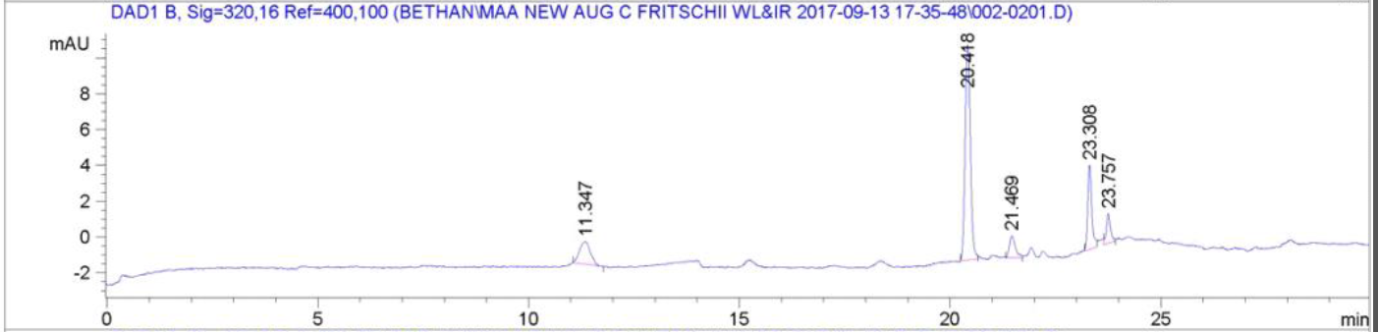
**

A

**
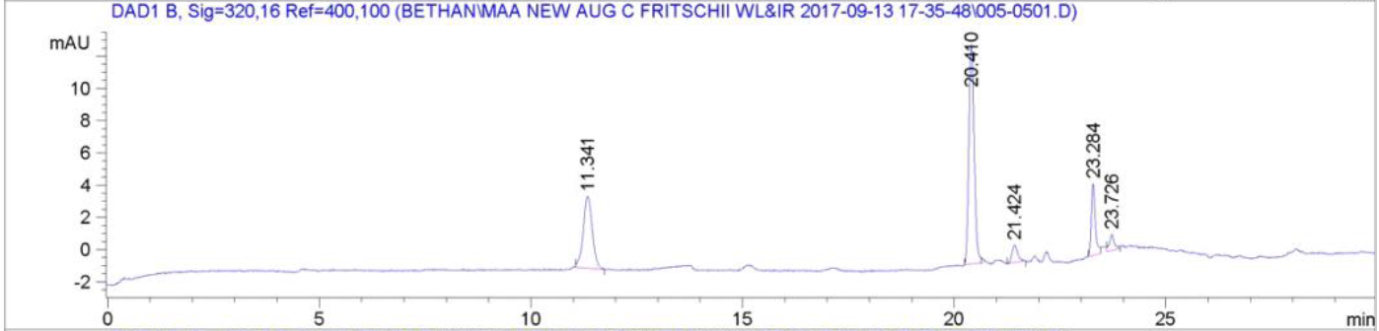
**

Retention Time (mins)

B

Representative HPLC chromatograms of extracts of MAAs from *C. fritschii* PCC 6912 exposed to white and FR light. Peak at 11.3 mins identified as shinorine (λ_max_ 334nm) and at 20.4 mins is mycosporine-glycine (λ_max_ 310nm). **A.** White light. **B.** FR light.

**Fig. S3**.

**
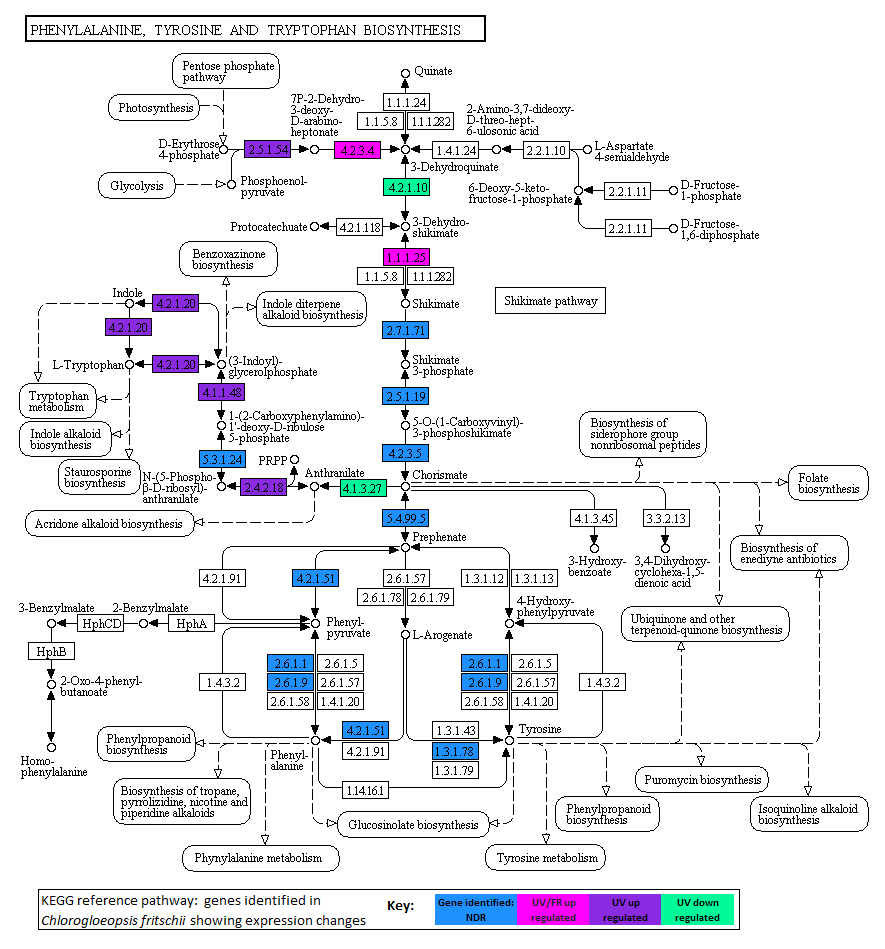
**

KEGG biosynthesis pathway for aromatic amino acids in *C. fritschii* PCC 6912 exposed to far-red and UV-B light.

**Fig. S4.**


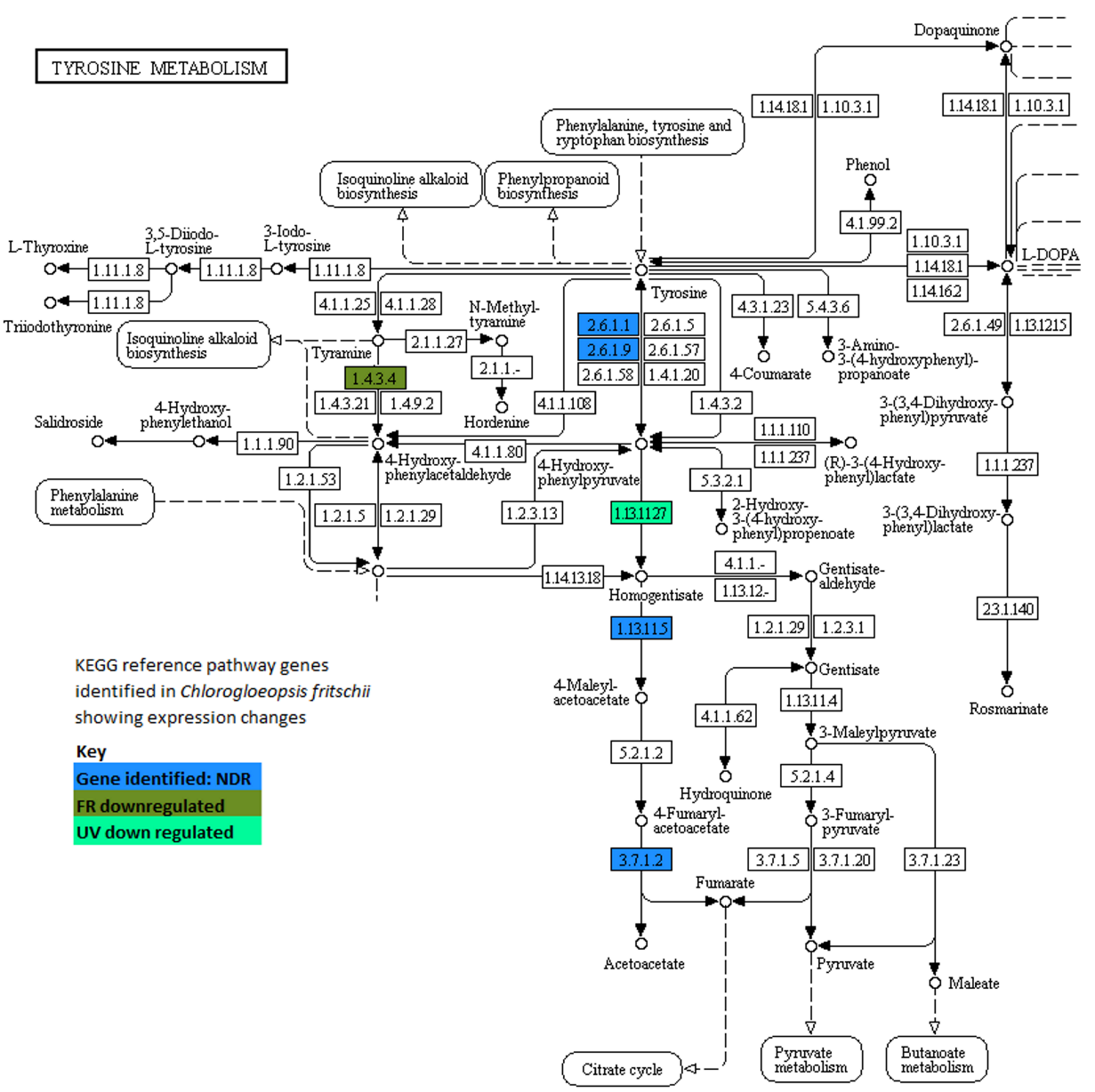


KEGG pathway for tyrosine metabolism in *C. fritschii* PCC 6912 exposed to far-red and UV-B light.

**Fig. S5.**


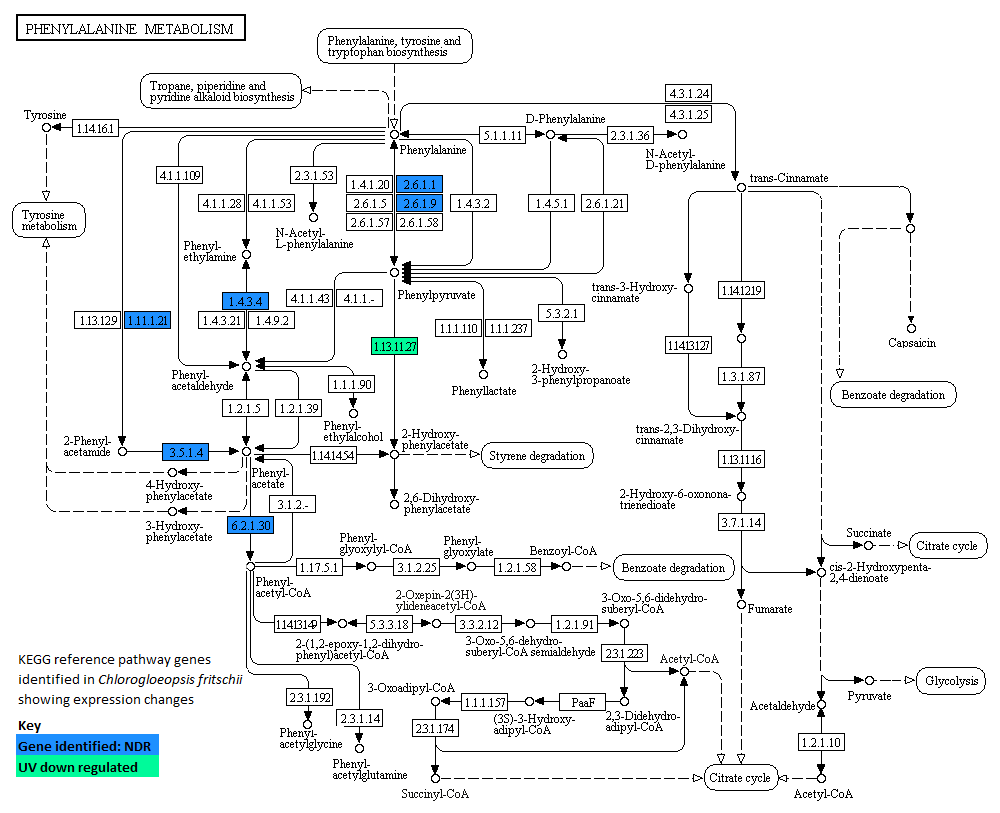


KEGG pathway for phenylalanine metabolism in *C. fritschii* PCC 6912 exposed to far-red and UV-B light.

**Fig. S6.**


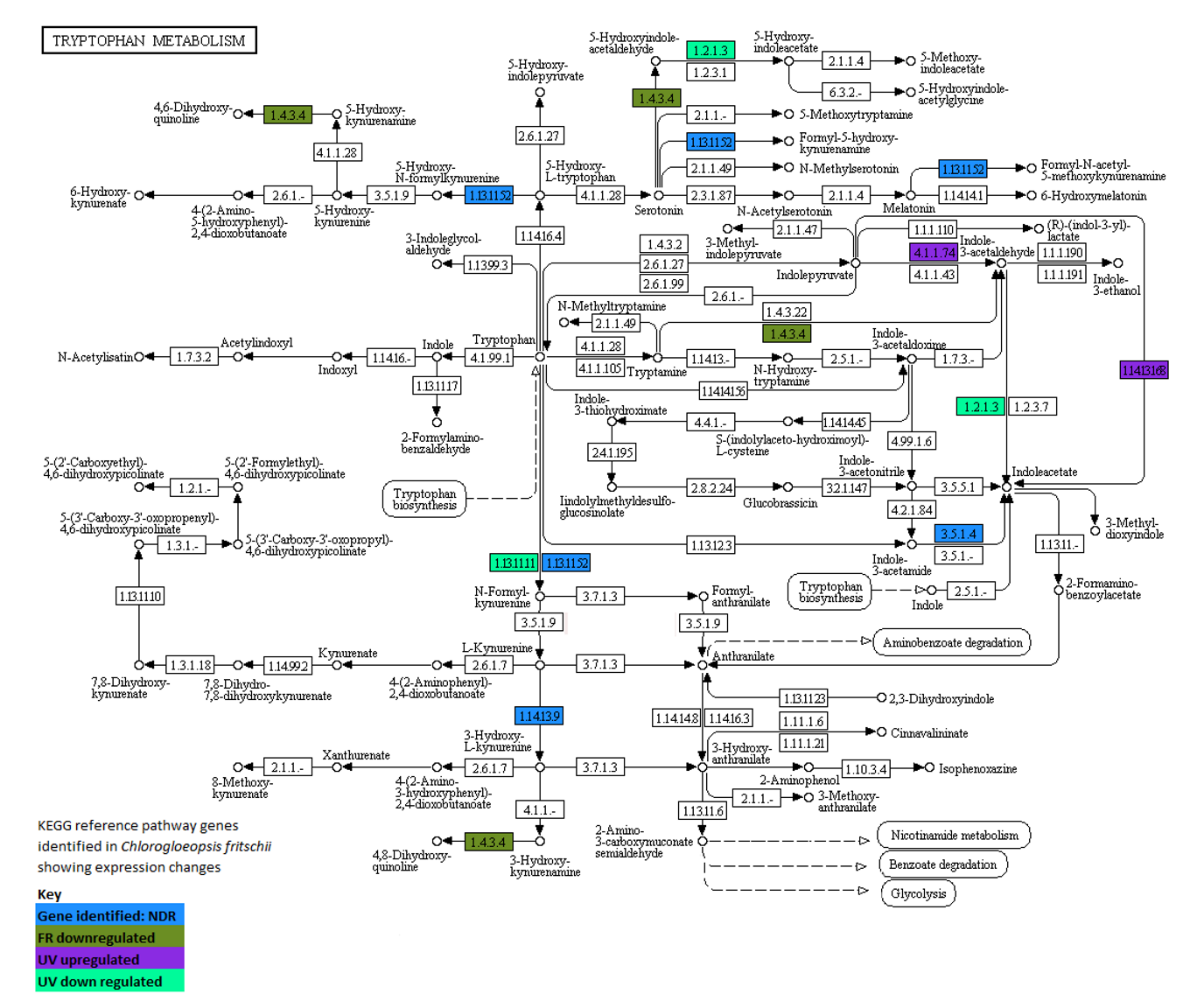


KEGG pathway for tryptophan metabolism in *C. fritschii* PCC 6912 exposed to far-red and UV light.
